# Using the Super Learner algorithm to predict risk of major adverse cardiovascular events after percutaneous coronary intervention in patients with myocardial infarction

**DOI:** 10.1186/s12874-024-02179-5

**Published:** 2024-03-08

**Authors:** Xiang Zhu, Pin Zhang, Han Jiang, Jie Kuang, Lei Wu

**Affiliations:** 1 Jiangxi Provincial Key Laboratory of Preventive Medicine, School of Public Health, Nanchang University, 461 BaYi St, Nanchang, 330006 People’s Republic of China; 2 School of Public Health and Management, Nanchang Medical College, Nanchang, People’s Republic of China; 3 Department of Cardiology, Second Affiliated Hospital of Nanchang University, Nanchang, 330006 People’s Republic of China

**Keywords:** Ensemble learning, Super Learner, Myocardial infarction, Percutaneous coronary intervention, Major adverse cardiovascular events

## Abstract

**Background:**

The primary treatment for patients with myocardial infarction (MI) is percutaneous coronary intervention (PCI). Despite this, the incidence of major adverse cardiovascular events (MACEs) remains a significant concern. Our study seeks to optimize PCI predictive modeling by employing an ensemble learning approach to identify the most effective combination of predictive variables.

**Methods and results:**

We conducted a retrospective, non-interventional analysis of MI patient data from 2018 to 2021, focusing on those who underwent PCI. Our principal metric was the occurrence of 1-year postoperative MACEs. Variable selection was performed using lasso regression, and predictive models were developed using the Super Learner (SL) algorithm. Model performance was appraised by the area under the receiver operating characteristic curve (AUC) and the average precision (AP) score. Our cohort included 3,880 PCI patients, with 475 (12.2%) experiencing MACEs within one year. The SL model exhibited superior discriminative performance, achieving a validated AUC of 0.982 and an AP of 0.971, which markedly surpassed the traditional logistic regression models (AUC: 0.826, AP: 0.626) in the test cohort. Thirteen variables were significantly associated with the occurrence of 1-year MACEs.

**Conclusion:**

Implementing the Super Learner algorithm has substantially enhanced the predictive accuracy for the risk of MACEs in MI patients. This advancement presents a promising tool for clinicians to craft individualized, data-driven interventions to better patient outcomes.

## Introduction

Percutaneous coronary intervention(PCI) has been suggested to be the primary treatment for patients with myocardial infarction [1–3], and its technical means have become increasingly mature [4]. Despite extensive progress in the field of interventional therapy, the incidence of major adverse cardiovascular events (MACEs) in patients with acute myocardial infarction (AMI) is still high [5]. A retrospective study of 15,009 patients with AMI showed that approximately 5.9% of patients with AMI developed MACEs [6]. However, PCI is still accompanied by various complications, such as bleeding, reflux, and thrombosis [7]. This will not only cause the failure of PCI to treat the original diseases, but may also lead to re-occurrence of myocardial infarction(MI) and death and other serious consequences. As such, tools available to assist clinicians in predicting events before they occur have vital utility in managing the health of MI patients.

At the same time, with the gradual improvement of the hospital information management system, the information platform of the hospital has formed a large amount of real world data. Real world data is defined as data relating to patient health status and/or the delivery of healthcare routinely collected from a variety of sources [8]. Martin Anderson further assessed the similarities between clinical trials and the real world population by comparing clinical trials with real world data for comparative analysis of peak inspiratory flow rates in patients with COPD [9].

Despite the continuous improvement in the quantity and quality of clinical patient data, the current status of research on prognosis outcome prediction of PCI for patients with myocardial infarction is still not optimistic. Almost all PCI prediction models are based on single models such as Cox regression and artificial neural network analysis, and validation is generally limited. A Japanese study screened out seven risk factors of acute kidney injury in patients after PCI by Lasso and SHAP methods, and applied the light GBM and logistic regression to construct prediction models, while the AUC of light GBM and logistic regression were 0.772 and 0.755 respectively [10]. Jacob A Doll adopted six machine learning methods, but the fitting results of each model were uneven [11]. As a result, another part of machine learning (ensemble learning) arises at the historic moment. Ensemble learning trains multiple machine learners through a certain combination strategy, and finally obtains a model with stronger learning ability [12]. Up to now, ensemble learning in machine learning [13–16] has become a priority in the establishment of prediction models based on PCI.

Among them, Super Learner(SL) algorithm [17] proposed by van der Laan integrates and learns multiple classical models such as random forest, artificial neural network and support vector machine by virtue of stack generalization principle and ensures the stability of the prediction model through cross validation. Compared with some single and emsemble prediction models, its risk prediction ability and generalization ability are significantly improved [18]. Multiple studies have shown that the predictive ability of the SL model ultimately formed in the fields of postpartum infection, disease onset and emotional disorders is significantly superior to the single model [19–21]. In addition, Super Learner has performed well in disease burden estimation in epidemiology [22].

Therefore, we constructed an ensemble learning model for PCI prognosis by collecting real world data of patients after PCI and screening out the risk factors that affect the incidence of MACEs after PCI by using Super Learner. The present study was to to explore the best model combination that accords with the prognosis of PCI and validate a Super Learner prediction model to predict risk of 1-year MACEs after percutaneous coronary intervention in patients with MI.

## Materials and methods

This study conformed to the TRIPOID(Transparent Reporting of a multivariable prediction model for Individual Prognosis or Diagnosis) reporting guidelines [23].

### Source of data

The research data were retrospectively collected from a large comprehensive medical institution( the Second Affiliated Hospital of Nanchang University) by two researchers. The relevant information of the patients (general information, medical history, blood test, and PCI related information) was obtained from the Hospital Management Information System (HIS).

### Participants

All patients who met the criteria of PCI and underwent PCI for MI were included from January 2018 to December 2021. The classification criteria for disease diagnosis were based on ICD-10 classification. The surgical indications of PCI include non-ST-segment elevation myocardial infarction, and acute ST-segment elevation myocardial infarction [24]. Patients with incomplete medical records, a history of PCI treatment or complications due to other heart conditions were excluded [25].

### Outcome

The primary outcome was 1-year MACEs. MACEs [26] mainly includes cardiac death, myocardial infarction, angina pectoris attack, heart failure, revascularization, malignant arrhythmia, stent thrombosis, etc. MACEs were obtained through follow-up by trained investigators.

### Predictor characteristics

Age, sex, Body Mass Index(BMI), pulse(The number of arterial beats per minute in the patient at rest), Killip classification, Previous disease (hypertension, hyperlipidemia, diabetes, renal insufficiency, pulmonary infection), systolic and diastolic blood pressure, smoking, ETOH abuse, family medical history (diabetes, hypertension, coronary heart disease), number of hospitalizations, number of diseased coronary arteries, electrocardiogram (sinus rhythm, atrial fibrillation, pacing rhythm, high or III degree atrioventricular block, ST segment changes, complete left bundle branch block, complete right bundle branch block, abnormal Q wave, left ventricular high voltage, and T-wave change), blood test (B-type natriuretic peptide(BNP), aspartate transaminase(AST), creatine kinase(CK), creatine kinase isoenzyme(CKMB), serum creatinine(Scr), estimated glomerular filtration rate(eGFR), and potassium(K)), vascular stenosis degree (left main shaft(LMA), left anterior descending branch(LAD), left circumflex branch(LCx), and right crown(RCA)), thrombolysis in myocardial infarction (TIMI) blood flow classification (LMA, LAD, LCx, and RCA), PCI information (cardiac arrest, time from onset to PCI, intervention approach, surgical method, and number of stents implanted) were included in the study as explanatory variables. The index information comes from the hospital information system and is obtained by professional clinicians.

Data analysis and sample size were performed using R software version 4.2.1 (R Foundation for Statistical Computing, Vienna, Austria). The missing values of continuous variables were filled by predictive mean matching, while the classified variables were filled by classification regression tree method. The sample size is calculated by using the pmsampsize function in R. Descriptive statistics were presented as median and quartile spacing or number and percentages for continuous and categorical variables, respectively.

### Model building and validation

Super Learner [17] is an estimator based on loss function, which combines multiple parametric, semi-parametric models or other appropriate models through multi-fold cross-validation. First, Super Learner automatically selects the function form of the initial candidate prediction model according to the provided data, and uses the loss function (mean square error, MSE) to evaluate the candidate model and the combination model. At the same time, different weights are given to each model through the coefficients to obtain an optimal combination model. Super Learner [27] includes multiple models, such as artificial neural network [28], recursive partition tree [29], support vector machine [30], random forest [31], extreme random tree [32], Xgboost [33], generalized additive model [34] and gradient boosting machines(gbm) [35], etc.

We had included the following kinds of algorithms in the SL model: Classification and Expression Training (caret) [36], RandomForest, conditional inference trees (cforest), multivariate adaptive regression splines (earth), generalized linear model (glm), Generalized additive model (gam), AIC stepwise regression (step), ridge regression (ridge), regularization regression (glmnet), Xgboost algorithm, bagging algorithm (ipredbagg), gradient boosting machines(gbm), non-negative least squares regression (nnls), support vector machine (svm), linear regression model (lm).

**Fig. 1:**
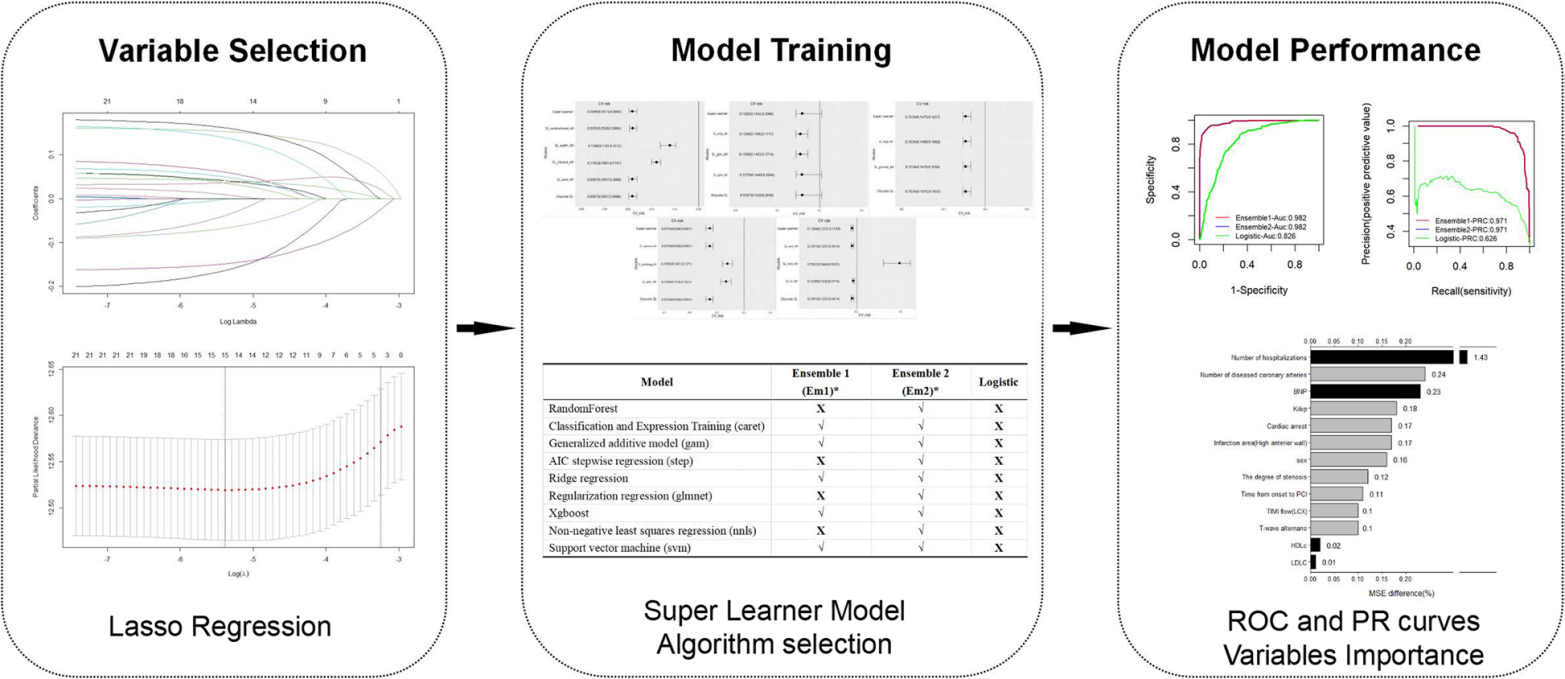
Workflow diagram

The model structure was further simplified by screening with lasso regression variables(Eliminate the variables with coefficient of 0 in the model), and the training set and test set were divided according to the proportion of 75% [37]. The Super Learner models with different combinations were trained based on the fivefold cross validation and the ROC curve and PR curve were drawn for model evaluation. The importance of explanatory variables was calculated by the MSE of the model after the explanatory variables were eliminated one by one (Fig. 1).

## Results

### Patients’ characteristics

We collected 4167 patients after PCI, excluding 287 patients who lost follow-up, and finally collected 3,880 patients who underwent PCI. Characteristics of patients are presented in Table 1: the follow-up patients with 1-year MACEs accounted for 12.2%, with age of 65 (57 to 72)years. Male patients numbered 2862 (73.8%) and a few suffered from hyperlipidemia (28.8%), diabetes (29.7%) and renal insufficiency (13.5%). Some patients had the habit of smoking (34.1%) and ETOH abuse (24.0%). There were fewer patients with family history. The results of electrocardiogram(ECG) showed that 95.4% of the patients had sinus rhythm, and most of them had Killip II (45.7%) and III (38.9%). In the 1-year MACEs group, patients had a higher proportion of diabetes and abnormal Q wave, a lower proportion of smoking, more diseased coronary artery branches and number of implanted stents, higher levels of BNP and Cre, and lower level of eGFR.

**Table 1:** Baseline characteristics

Variable	Total (*n* = 3880)	1-year MACEs (*n* = 475)	nonMACEs (*n* = 3405)
**Age(Years)**, median(IQR)	65(57 to 72)	66(59 to 73)	65(57 to 72)
**Sex(male)**, n(%)	2862(73.8)	364(76.6)	2498(73.4)
**Previous disease**, n(%)			
Hypertension	2220(57.2)	292(61.5)	1928(56.6)
Hyperlipidemia	1117(28.8)	127(26.7)	990(29.1)
Diabetes	1151(29.7)	165(34.7)	986(29.0)*
Renal insufficiency	523(13.5)	78(16.4)	445(13.1)
Pulmonary infection	353(9.1)	59(12.4)	294(8.6)
**Smoking**,n(%)	1324(34.1)	160(33.7)	1164(34.2)*
**ETOH abuse**,n(%)	933(24.0)	113(23.8)	820(24.1)
**Family medical history**, n(%)			
Diabetes	83(2.1)	10(2.1)	73(2.1)
Hypertension	161(4.1)	18(3.8)	143(4.2)
Coronary heart disease	71(1.8)	8(1.7)	63(1.9)
**Electrocardiogram**, n(%)			
Sinus rhythm	3701(95.4)	452(95.2)	3249(95.4)
Atrial fibrillation	172(4.4)	22(4.6)	150(4.4)
Pacing rhythm	374(9.6)	47(9.9)	327(9.6)
High or III degree atrioventricular block	76(2.0)	11(2.3)	65(1.9)
ST segment change	1623(41.8)	198(41.7)	1425(41.9)
Complete left bundle branch block	60(1.5)	8(1.7)	52(1.5)
Complete right bundle branch block	225(5.8)	31(6.5)	194(5.7)
Abnormal Q wave	1058(27.3)	133(28.0)	925(27.2)*
Left ventricular high voltage	545(14.0)	71(14.9)	474(13.9)
T-wave alternans	1131(29.1)	143(30.1)	988(29.0)
**Killip classification**, n(%)			
I	249(6.4)	27(5.7)	222(6.5)
II	1772(45.7)	219(46.1)	1553(45.6)
III	1508(38.9)	197(41.5)	1311(38.5)
IV	154(4.0)	19(4.0)	135(4.0)
**Number of hospitalizations**, median(IQR)	2(2 to 3)	2(2 to 3)	2(2 to 3)
**Number of diseased coronary arteries**, median(IQR)	2(2 to 3)	3(2 to 3)	2(2 to 3)*
**BMI(kg/m**^**2**^**)**, median(IQR)	23.9(22.0 to 25.7)	23.8(21.9 to 25.7)	23.9(22.0 to 25.7)
**Pulse(bpm)**, median(IQR)	87(80 to 94)	88(79 to 94)	87(80 to 94)
**SBP(mmHg)**, median(IQR)	129(115 to 145)	128(115 to 145)	129(115 to 145)
**DBP(mmHg)**, median(IQR)	70(63 to 77)	70(63 to 77)	70(63 to 77)
**Blood test**, median(IQR)			
BNP(pg/mL)	150.3 (50.0 to 445.7)	223.7(75.6 to 591.9)	142.9(48.4 to 423.3)*
AST(U/L)	28.3(21.2 to 54.1)	27.6(21.1to 50.8)	28.4(21.2 to 54.2)
CK(U/L)	126.5(82.0 to 317.9)	127.1(80.5 to 376.9)	126.5(82.3 to 315.2)
CKMB(U/L)	19.8(13.7 to 39.9)	19.7(13.5 to 41.0)	19.8(13.7 to 39.7)
Cre(μmoI/L)	81.8(68.6 to 99.7)	87.2(71.8 to 106.3)	81.4(68.1 to 98.7)*
eGFR(ml/ (min × 1.73m^2^))	82.4(65.1 to 99.0)	76.3(59.4 to 95.8)	83.1(65.8 to 99.5)*
Scr(mg/dL)	66.9(48.0 to 87.5)	61.5(42.7 to 76.0)	67.8(48.7 to 88.7)
K(mmol/L)	3.9(3.6 to 4.2)	3.9(3.7 to 4.2)	3.9(3.6 to 4.2)
**PCI surgery information**,n(%)			
Cardiac arrest	58(1.5)	11(2.3)	47(1.4)
Time from onset to PCI			
< 3 h	189(4.9)	23(4.8)	166(4.9)
3-6 h	202(5.2)	25(5.3)	177(5.2)
6-9 h	362(9.3)	56(11.8)	306(9.0)
9-12 h	359(9.3)	45(9.5)	314(9.2)
> 12 h	2630(67.8)	301(63.4)	2329(68.4)
Intervention approach			
Femoral artery	178(4.6)	34(7.2)	144(4.2)
Radial artery	3676(94.7)	436(91.8)	3240(95.2)
Ulnar artery	26(0.7)	5(1.1)	21(0.6)
Surgical method			
Thrombus aspiration	44(1.1)	8(1.7)	36(1.1)
PTCA	3595(92.7)	445(93.7)	3150(92.5)
Stent placement	241(6.2)	22(4.6)	219(6.4)
Number of stents implanted	2(1 to 2)	2(1 to 3)	1(1 to 2)*

^*^P-value < 0.05

### Prediction model development

#### Variable and model selection

**Fig. 2:**
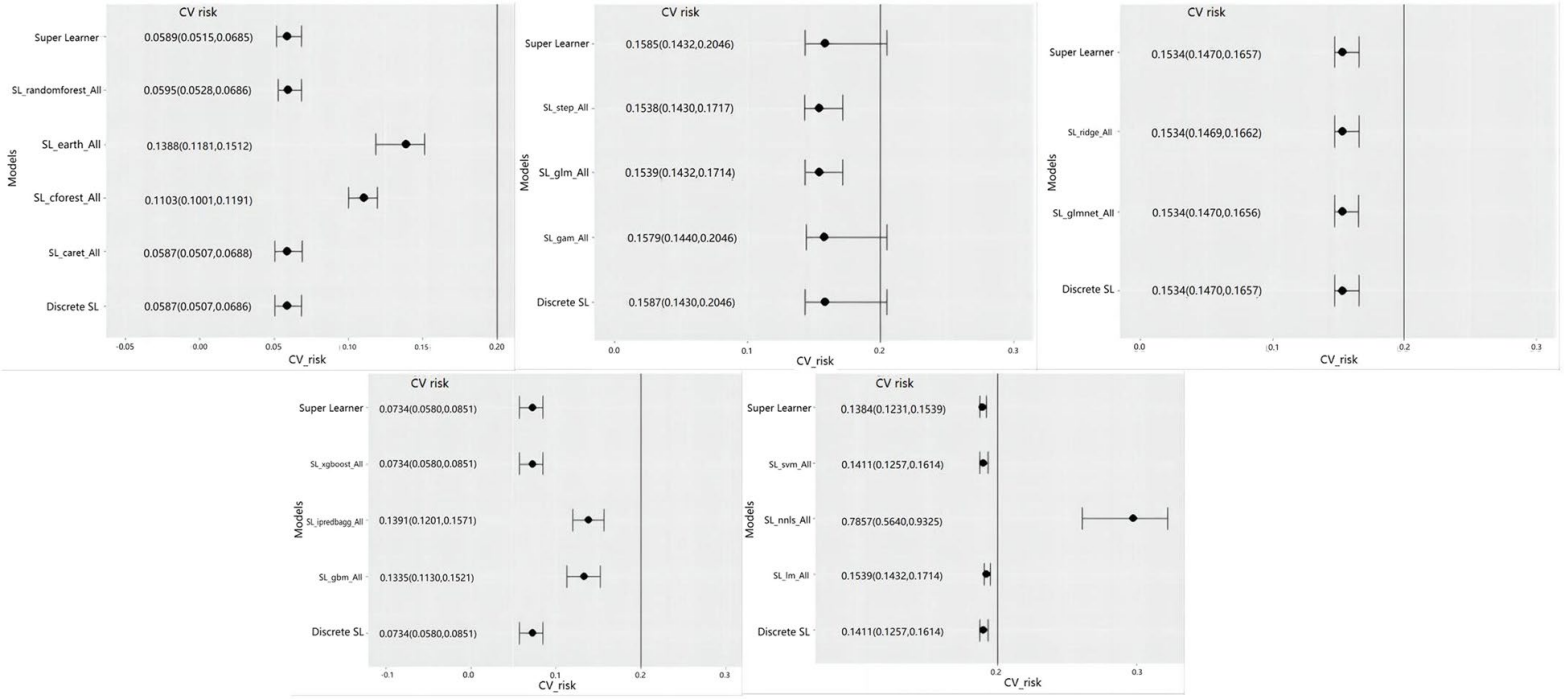
Cross validation risk map of training model (*n* = 3751)

With 1-year MACEs as outcome variables, variables were included in lasso regression, and 13 variables that affected the outcome were screened out and included in the SL model. Since the proportion of outcome variable was too small and belonged to unbalanced data [38], the data were subjected to over-sampling processing, and the sample size finally included in the model was 5000 cases. Then the training set (*n* = 3751) and the test set (*n* = 1249) were randomly divided, and different hybrid models (Model 1: caret, RandomForest, cforest, earth; Model 2: glm, gam, and step; Model 3: ridge, glmnet; Model 4: Xgboost, ipredbagg, gbm, and Model 5: nnls, svm, and lm). The five models were trained one by one through the training set. The cross-validation risks and confidence intervals of the five models are shown in Fig. 2. The models with nonzero coefficients in Model 1 are RandomForest (coef = 0.173) and caret (coef = 0.827). The models with nonzero coefficients in model 2 were gam (coef = 0.618) and step(coef = 0.382), the model with nonzero coefficients in model 3 were ridge (coef = 0.622) and glmnet(coef = 0.378), the models with nonzero coefficients in model 4 was only Xgboost, and the models with nonzero coefficients in model 5 were nnls (coef = 0.141), svm (coef = 0.859).

#### Model performance

**Fig. 3:**
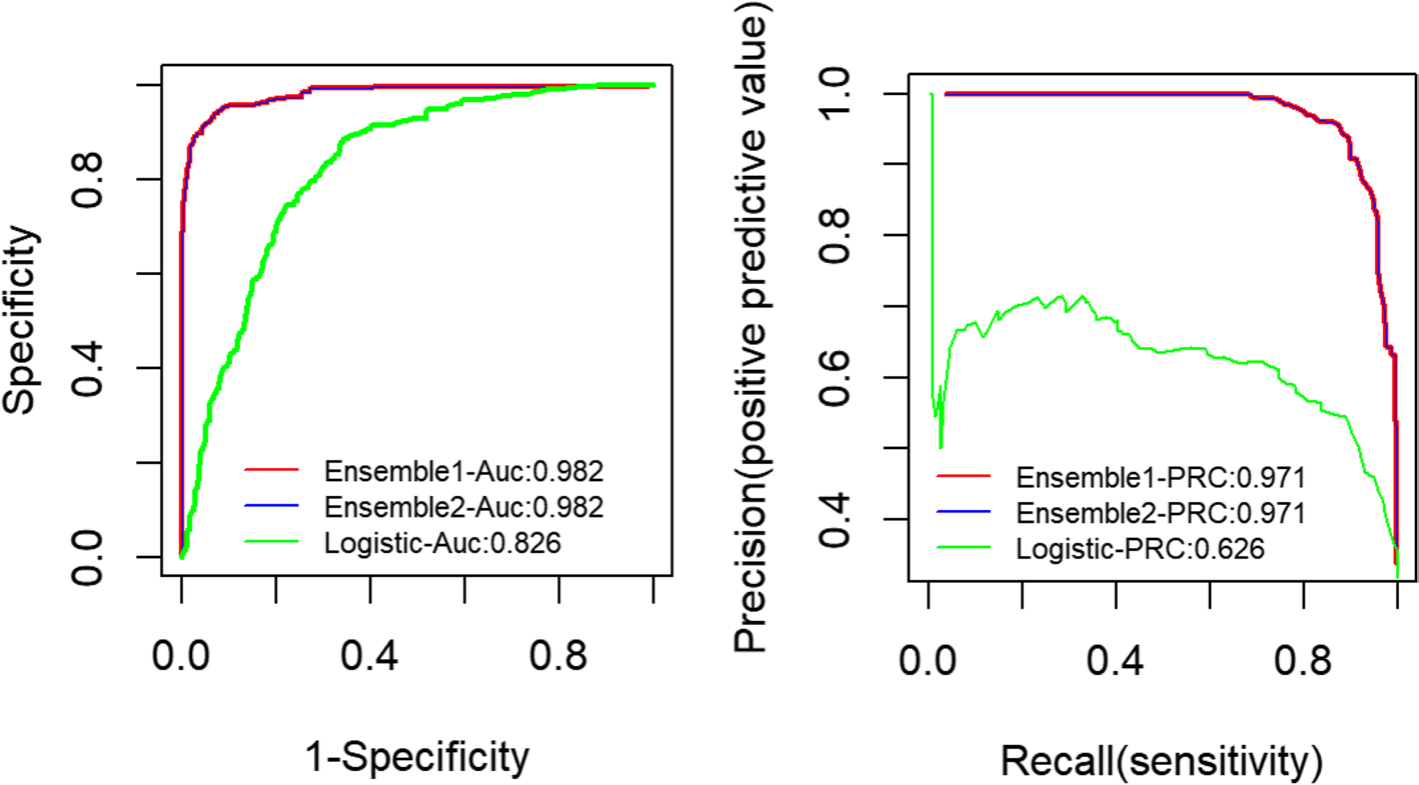
ROC and PR of ensemble model (*n* = 1249)

According to the representation of the training set on each hybrid model, two ensemble models were established. Em1 and Em2 were respectively the single model combination with the highest coefficient (caret, gam, ridge, Xgboost and svm) and nonzero coefficient (RandomForest, caret, gam, step, ridge, glmnet, Xgboost, nnls and svm). The specific single models included in the ensemble model are shown in Table 2. The predictive abilities of the four ensemble models were tested on the test set and the ROC and PR plots were drawn. The results showed that all the models performed well on the test set, with AUC ranging from 0.826 to 0.982, and AP ranging from 0.626 to 0.971. Em1 and Em2 showed the best performance (AUC:0.982 (95% CI: 0.975–0.989) and AP:0.971(95% CI: 0.947–0.994)), which was significantly higher than that of logistic regression (AUC:0.826 (95% CI: 0.804–0.848)and AP:0.626(95% CI: 0.602–0.650)) (Fig. 3). The confusion matrix results showed that Em1 and Em2 had the same accuracy(0.922, 95% CI: 0.906–0.937), the sensitivity of Em1 was the highest (0.907) and the specificity of Em2 was the highest (0.957).In addition, the Kappa of Em1 and Em2 both were both above 0.8.

**Table 2:** Basic learning algorithm contained in each integration model (*n* = 3751)

Model	Ensemble 1 (Em1)	Ensemble 2 (Em2	Logistic
RandomForest	**X**	**√**	**X**
Classification and Expression Training (caret)	**√**	**√**	**X**
Generalized additive model (gam)	**√**	**√**	**X**
AIC stepwise regression (step)	**X**	**√**	**X**
Ridge regression	**√**	**√**	**X**
Regularization regression (glmnet)	**X**	**√**	**X**
Xgboost	**√**	**√**	**X**
Non-negative least squares regression (nnls)	**X**	**√**	**X**
Support vector machine (svm)	**√**	**√**	**X**

#### Variable importance

**Fig. 4:**
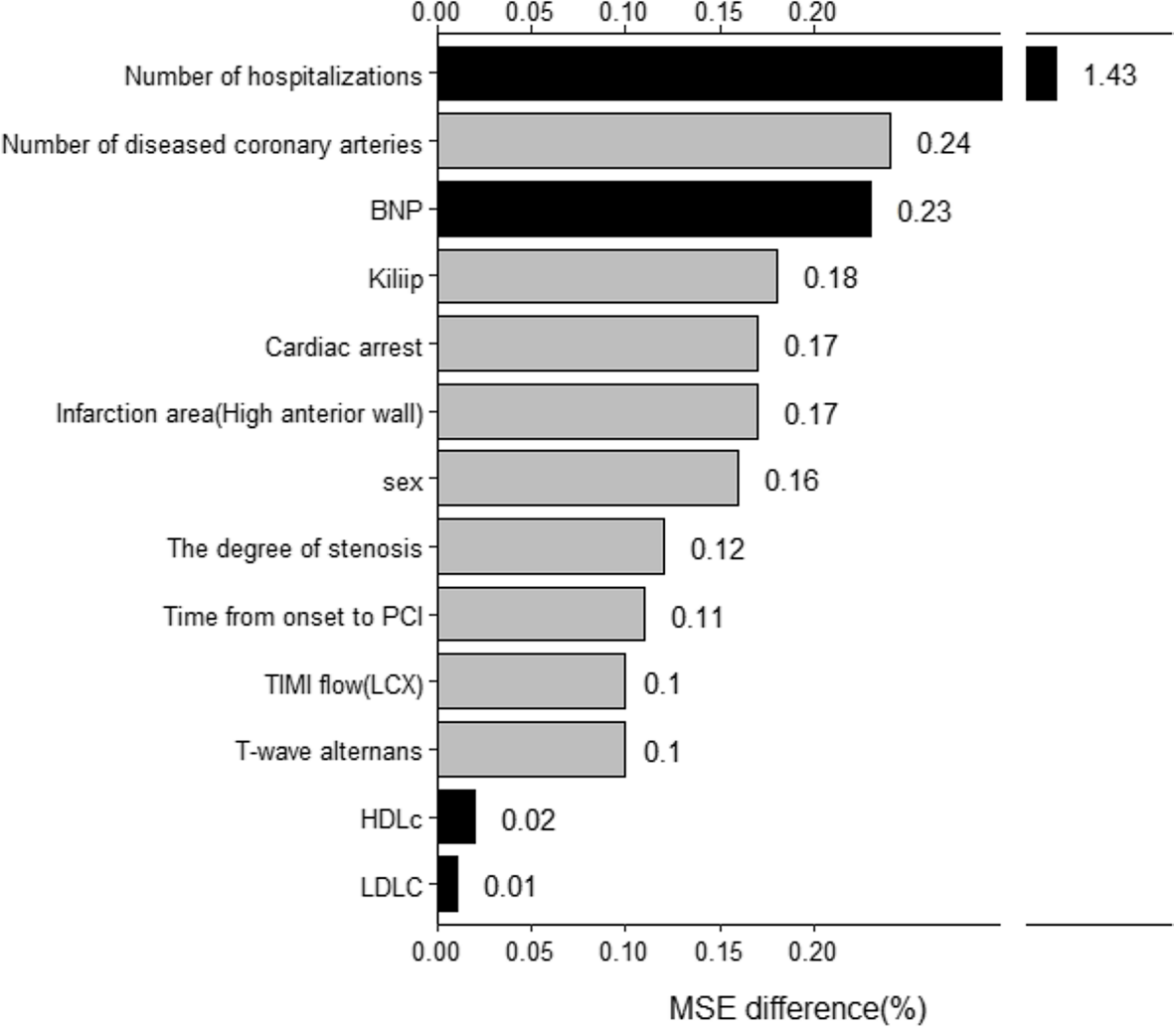
Importance ranking of Em1 variables (A grey bar indicates a positive correlation and black indicates a negative correlation with the SL-predicted score)

The importance of each included variable was calculated and sorted by eliminating the variables one by one. For Em1, the most important factors were the number of hospitalizations(1.43% MSE difference), number of diseased coronary artery(0.24% MSE difference), BNP(0.23% MSE difference), Killip(0.18% MSE difference), and cardiac arrest(0.17% MSE difference) (Fig. 4). Both sets of models considered that the number of hospitalizations is the most important predictor(MSE > 1). It showed that the number of hospitalizations is the most influential factor in the occurrence of MACEs after PCI. The number of diseased coronary artery, Killip, and cardiac arrest were positively correlated with the predicted value of 1-year MACEs. And the number of hospitalizations and BNP were negatively correlated with the prediction of 1-year MACEs.

## Discussion

The present study used a large population-based clinical database and machine learning with ensemble learning. Our results showed that 12.2% of the follow-up patients with PCI developed MACEs in a year.We also found that the most appropriate and simplest model combination for PCI prognosis is Em1 (caret, gam, ridge, Xgboost and svm). The SL predictive model established for MACEs in a year after PCI showed good performance (AUC: 0.982, AP: 0.971).The model relied on the integration of multiple models and used real world data from hospital systems.

In fact, 1-year MACEs has become a common index to evaluate the prognosis of PCI. The definition of MACEs includes stent thrombosis, cardiac death, myocardial infarction, and all-cause death [39]. Obviously, compared with the simple postoperative mortality and readmission rate, MACEs include most of the adverse prognosis of patients with MI after PCI, which is very important to evaluate the surgical treatment. Several studies [39, 40] have shown that 1-year MACEs is significantly more accurate in evaluating the prognosis of patients with PCI than other indicators. For this reason, the present study aims to develop a SL model to explore the related risk factors for 1-year MACEs after PCI. Our model ultimately included 3880 patients, of which 475 (12.2%) developed MACEs within 1 year. The incidence of 1-year MACEs is similar to data recently in other study [41].

Various machine learning models have been applied to predict MACEs in the prognosis studies of PCI. A retrospective cohort study from New York used the adjusted Cox regression model to assess the effect of high-sensitivity C-reactive protein on MACEs after PCI [42]. Another prospective study in China compared and analyzed six different models (svm, decision tree, RandomForest, gradient-based decision tree, neural network, and logistic regression) for predicting the long-term prognosis of PCI [43]. Besides, a prediction model based on an artificial neural network showed that the accuracy in the test set was more than 80% [44]. Different models have different effects on predicting the prognosis of PCI and have their own advantages and disadvantages. Ensemble learning combines the advantages of each single model with the hybrid learning of a single model, thus effectively improving the accuracy and applicability of the prediction model. Multiple studies had shown that an ensemble machine learning model is often superior to a single prediction model [45, 46]. And Super Learner belongs to stacking generalization in integration method, that is, combining several different prediction model algorithms into an integrated model, and then using V-fold cross validation to construct the optimal weighted combination of prediction from the candidate algorithm library, thus improving the prediction accuracy of the final model [47]. In fact, some studies have confirmed that Super Learner performs well in both survival prediction [48] and disease severity prediction [49]. Compared with some models related to the prognosis of PCI [13–16], our research found that the most suitable combination of PCI prognosis prediction models mainly includes caret, gam, ridge, Xgboost and svm. And compared with the traditional logistic regression model, the predictive performance of that ensemble learning model in the test set is sufficient to indicate the application value of the Super Learner(AUC:0.982 (95% CI: 0.975–0.989) and AP:0.971(95% CI: 0.947–0.994)).

Our study determined that the number of hospitalizations was an important risk factor for MACEs after PCI. Our findings align with Sinjini, which observed a relationship between the number of hospitalizations and the occurrence of heart disease after PCI [50]. The reasons for such an association are likely multifactorial. May be one or more hospitalizations before cardiac problem treatment due to different diseases, and these previous disease histories also cause changes in patients' health conditions and increase the recurrence rate of cardiac problems after discharge, thus increasing the risk of readmission [51–53]. In addition, Grace Dibben summarized that with the increase in the number of hospitalizations, the exercise time of patients with heart disease decreased, resulting in an increased risk of myocardial infarction and greatly improved all-cause hospitalization and small increase in all-cause mortality [54].

BNP has also been found to be a risk factor for the prognosis of PCI. The conclusions of our study are consistent with those of many studies [55–59]. As a hormone secreted by the heart, BNP has been proved to have multiple effects. In the kidney, they increase glomerular filtration and inhibit sodium reabsorption, causing natriuresis and diuresis. For cardiovascular, BNP can relax vascular smooth muscle, causing arterial and venous dilatation, and resulting in decreased blood pressure and ventricular preload. Moreover, a meta-analysis confirmed the predictive power of BNP for postoperative major adverse cardiac events. And the heart risk index is remarkably improved after the BNP index is increased [60]. A clinical randomized controlled trial of patients who successfully underwent revascularization showed that compared with placebo, patients who received BNP injection and had a baseline left ventricular ejection fraction of < 40% tended to reduce the size of left ventricular infarction [61]. In addition, compared with conventional risk factors and other markers of arterial compliance, inflammation and autonomic nerve function, BNP has a higher value in predicting the outcomes of patients with altered risk of coronary artery disease, and is more capable of independently identifying patients with slightly impaired cardiac function [62].

Compared with the previous study [63], we also found an additional correlation between the number of diseased coronary artery and the occurrence of MACEs. In fact, as early as the twentieth century, X Bosch have proved that patients with myocardial infarction with more diseased coronary arteries are more likely to have early ischemia [64]. However, for patients undergoing percutaneous transluminal coronary angioplasty, the number of diseased coronary segments with stenosis greater than 70% is the most important parameter affecting the outcome of patients [65]. Instead, several studies [66] have not confirmed the relationship between the number of diseased coronary arteries and cardiovascular adverse events. We believe that there may be several reasons. First, this randomized trial included patients with multi-vessel coronary artery disease and patients with ST-segment elevation myocardial infarction, and did not analyze the number of diseased coronary artery. However, the patients included in our study were patients with myocardial infarction. Patient-to-patient comparability may be greater by collecting the number of diseased coronary arteries for analytical comparison. Second, there are differences in outcome indicators for comparison. The prognostic outcome of our study was 1-year MACEs, while the main cardiovascular and cerebrovascular adverse events (MACCE) in this randomized controlled trial were collected.

The main advantages of this study lie in that the patient data were obtained from the medical record system of medical institutions, while the prediction model constructed based on the real world data could be better applied to clinical practice. Compared with randomized controlled trials with more stringent inclusion and exclusion criteria, clinical evidence formed from real world data can explore the disease characteristics in the real diagnosis and treatment environment, understand the patient size, disease burden, clinical characteristics and treatment mode in the real target population, and provide important evidence for the evaluation of the clinical value of the prediction model. Therefore, compared with the research conducted by Shi B et al [67], our research results are more suitable for scoring construction in the Asian population. In addition, another benefit of this study is the reporting that followed the TRIPOID statement.

Some limitations should be mentioned. First, the collection of case data of the study is conducted in a medical institution with extensive experience in PCI treatment. Although Super Learner allows the model to establish internal verification and conduct five-fold cross-verification, the results of this research still lack external verification. Second, most of the variables included in the study were pre-PCI examination data, and no detailed analysis of post-PCI examination data was conducted and included in the study. Whether the changes in the values of post-PCI examination data have an impact on the occurrence of 1-year MACEs remains to be discussed. Finally, although Super Learner improves the prediction performance of the prediction model, class-imbalance data and fewer observed events remain a limitation of this study. Therefore, a larger scale verification research should be carried out in the future to ensure the universality and stability of the algorithm.

## Conclusions

In conclusion, our study provide evidence of improved MACEs risk prediction and classification associated with the Super Learner algorithm in patients with MI, also highlighting the potential value of ensemble machine learning algorithms to improve risk prediction tools. These tools have the potential to aid clinicians to develop targeted interventions that may prevent an unnecessary MACEs.

## Data Availability

The datasets used and/or analyzed during the current study are available from the corresponding author on reasonable request.

## Abbreviations

AST: Aspartate aminotransferase
AUC: Area Under the Curve
CK: Creatine Kinase
CKMB: Creatinekinase-MB
Cre: Creatinine
CVD: Cardiovascular disease
ECG: Electrocardiograph
eGFR: Estimated glomerular filtration rate
HIS: Hospital information system
IQR: Interquartile range
LAD: Left anterior descending
Lcx: Left circumflex coronary artery
LM: Left main
MACCE: Main cardiovascular and cerebrovascular adverse events
MACEs: Major Adverse Cardiovascular Events
MI: Myocardial infarction
MSE: Mean Square Error
PCI: Percutaneous coronary intervention
PRC: Precision recall curve
PTCA: Percutaneous transluminal coronary angioplasty
RAAS: Renin–angiotensin–aldosterone system
RCA: Right coronary artery
ROC: Receiver Operating Characteristic
Scr: Serum creatinine
SL: Super learner
